# Nonbiodegradable Spiegelmer-Driven Colorimetric Biosensor for Bisphenol A Detection

**DOI:** 10.3390/bios12100864

**Published:** 2022-10-12

**Authors:** Shuo Ren, Samuel Cho, Ruixan Lin, Vinayakumar Gedi, Sunyoung Park, Chul Woo Ahn, Dong-Ki Lee, Min-Ho Lee, Sangwook Lee, Soyoun Kim

**Affiliations:** 1 PCL Inc., Seoul 05854, Korea; 2 School of Integrative Engineering, Chung-Ang University, Seoul 06974, Korea; 3 Global Research Laboratory (GRL) for RNAi Medicine, Department of Chemistry, Sungkyunkwan University, Suwon 16419, Korea; 4 Gangnam Biomedical Research Center, Yonsei University College of Medicine, Seoul 06273, Korea; 5 Division of Endocrinology Department of Internal Medicine, Yonsei College of Medicine, Seoul 03722, Korea; 6 Severance Institute for Vascular and Metabolic Research, Yonsei University College of Medicine, Seoul 03722, Korea; 7 Research Center for Advanced Science and Technology (RCAST), The University of Tokyo, Tokyo 153-8904, Japan; 8 Convergence Research Institute, Korea University, Seoul 02841, Korea

**Keywords:** spiegelmer, bisphenol A (BPA), L-DNAzyme, aptasensor, colorimetric assay

## Abstract

Spiegelmers are enantiomers of natural D-oligonucleotides that bind to targets with distinct structures such as aptamers. The high susceptibility of natural D-form aptamers to nucleases greatly hinders their application in biological environments. Here, a nonbiodegradable spiegelmer-based platform for the sensitive detection of bisphenol A (BPA) was developed. Due to the symmetric molecule of BPA, the D-form aptamer can be directly converted into mirror forms via chemical synthesis. Aptamer–target interactions that involve chemically synthesized spiegelmers were characterized by biolayer interferometry, and their stabilities were tested in various biological fluids by exposure to nucleases. We demonstrate for the first time the use of a nuclease-resistant spiegelmer in a simple, label-free gold nanoparticle-based colorimetric assay to detect BPA in a highly sensitive and selective manner. The aptasensor exhibits an LOD of 0.057 ng/mL and dynamic range of 10^5^ (100 pg/mL to 10 mg/mL). With sensing capacity and biological stability, the developed aptasensor shows great potential to utilize in in-field applications such as water quality monitoring.

## 1. Introduction

Many polycarbonate plastics contain bisphenol A (BPA), which is one of the most widely produced chemicals in the world. It is used in hard plastics, food cans, drink cans, receipts, and dental sealants. BPA is a small carcinogenic molecule (MW = 228 Da) linked to breast and prostate cancer [1,2]. BPA is regarded as an endocrine disruptor because it can mimic the action of hormone estrogen and disturb the estrogen–estrogen receptor binding process (hormonal pathways) [3,4]. Due to the threats it poses to the environment and human health, the need to detect and monitor BPA is increasing. Environmental monitoring requires a fast, low-cost and continuous analytical method for detection. Conventional detection technologies such as gas chromatography–mass spectrometry (GC–MS) and high-performance liquid chromatography (HPLC) provide accurate detection and a low range of the limit of detection (LOD) [5]. However, these techniques require complicated and laborious sample pretreatments, which necessitate a long working time for measurement, and they require bulky equipment, thereby rendering on-site and real-time monitoring difficult. An immune enzyme-based assay has been developed for rapid detection. However, the discovery of antibodies involves the chemical synthesis of an antigen conjugate, and the selection process is time consuming. Stringent assay conditions and possible antibody cross-activities reduce the detection accuracy. In particular, ELISA showed insensitive assay results because the BPA antibody exhibits nonspecific binding, especially for similar molecules, such as bisphenol B (BPB) or the analog 4,4-bis-(4-hydroxyphenyl) valeric acid [6].

Aptamers are single D-form oligonucleotides of 20 to 100 bp in length that have affinity and specificity against target molecules. They bind diverse targets that range from proteins and peptides to small organic/inorganic molecules with a similar affinity in the nanomolar to picomolar range compared with antibodies [7]. The advantages of an aptamer over an antibody include the lower molecular weight of the oligonucleotide and its fast chemical synthesis. Such advantages render aptamers superior to antibodies in applications such as biosensors, bioimaging, and therapeutics [8,9,10]. However, nuclease sensitivity is a major limitation of aptamers. Spiegelmers, which are chemically synthesized mirror-image oligonucleotides (L-configurations), represent a sophisticated approach to solving the biostability problem of aptamers due to their strong resistance to nuclease degradation. Typically, the systematic evolution of ligands by exponential enrichment (SELEX) [11] uses the synthetic D-oligonucleotide library (with a population of up to 10^15^) to identify aptamers. The SELEX process cannot be used directly to produce spiegelmers because there is no available enzyme for nucleic acid enrichment or reverse transcription. The general spiegelmer selection method is implemented according to the rules of symmetry: First, an aptamer is selected against the mirror form of the target. Once a suitable aptamer is identified, a mirror-form aptamer is chemically synthesized, as the original aptamer was selected for affinity against a mirror image of the target molecule [12]. Several spiegelmers were selected in various studies, and some were under clinical trials as drug candidates for diseases such as pancreatic, colorectal, and brain cancers, along with myeloma and leukemia [13,14]. Recently, L-DNAzyme- and L-DNA-templated nanoparticles or tetrahedron nanostructures were used for metal ion sensing and molecular imaging and as anticancer drug delivery vehicles [15,16,17].

**Figure 1:**
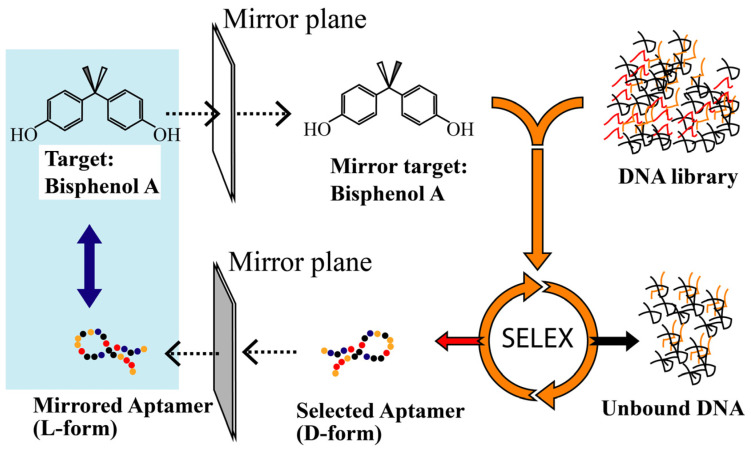
Illustration of the generation of a spiegelmer against the target. In the first step, a mirror-image form of the natural target is chemically synthesized (the original target is converted into a mirror-image target). Then, D-aptamers that bind to the mirror-image selection target are identified by in vitro selection from a synthetic oligonucleotide library in the natural d-configuration. The natural d-configuration is required because stereoselective enzymes are used for amplification, cloning and sequencing of bound sequences. In the second step, the enriched D-form library is sequenced, and the identified sequences are synthesized using enantiomeric L-oligonucleotides. If the principles of chirality are followed, the resulting L-aptamers (spiegelmers) will bind to the original natural target.

On the basis of the reciprocal chiral structure of BPA, we converted the D-form aptamer directly to its enantiomer, namely, an L-form as shown in Figure 1. The biostability of L-T4 (the L-conformation of the truncated D-form aptamer) [18] was tested against nucleases, and the binding affinities were characterized using biolayer interferometry. The cross-reactivity was analyzed against BPA analogs. Furthermore, L-T4 was utilized in two sensing platforms: nuclease-resistant and nuclease-sensitive colorimetric sensors that were developed based on gold nanoparticles and a competitive ELISA that utilizes biolayer interferometry.

## 2. Materials and Methods

### 2.1. Reagents

BPA (4,40 diphenylpropane; Sigma-Aldrich, MO, USA) was dissolved in 50% DMSO (dimethylsulfoxide, Sigma-Aldrich) at a final concentration of 20 mM. BPA analogs bisphenol B (BPB), 6F-bisphenol A (6F), and 4.4′-bisphenol (BP) were purchased from TCI (Tokyo, Japan). A stock solution of BPA was prepared by dissolving 1 mM BPA in BPA-binding buffer (25 mM Tris-HCl, 100 mM NaCl, 25 mM KCl, and 2 mM MgCl_2_; pH 7.4). BPA-4-HRP (bisphenol A-4-HRP, CosmoBio, Tokyo, Japan), anti-BPA antibody (CosmoBio, Tokyo, Japan), and a metal-enhanced DAB substrate kit (Thermo Fisher Scientific, Waltham, MA, USA) were used for a BLI-based BPA competition assay. Tris(hydroxymethyl)aminomethane hydrochloride, sodium chloride, potassium chloride, and magnesium chloride were purchased from Bioneer (Daejeon, Korea). All chemicals were of analytical reagent grade or better. All solutions were prepared using triple deionized distilled water (Milli-Q water purification system, Bedford, MA, USA) that was subsequently filtered using a 0.20 µm membrane filter system.

D-T4 (aptamer) and L-T4 (spiegelmer) were designed using the BPA#3 sequence from previous papers [18] and reprinted in the S-1. Both were synthesized by ChemGenes (Wilmington, MA, USA) either with or without 5′-biotin modification and purified by HPLC before use. Negative controlled nucleic acids such as oligo dT and TATA dsDNA) were synthesized by IDT (ChemGenes, Wilmington, MA, USA) and their sequences were also printed in S-1.

### 2.2. Determination of Dissociation Constants

The equilibrium dissociation constants (*K_D_*) of D-T4 and L-T4 against BPA were measured by the label-free biolayer interferometry technique using the same method as described in a previous publication [19].

### 2.3. Biostability Assays

The biostabilities of D-T4 and L-T4 were tested by Nuclease S1 (N 5661, Sigma, Burlington, MA, USA), TURBO^TM^ DNase (AM2238, Thermo Fisher Scientific, USA), and human serum (H6914, Sigma, MA, USA) digestion. D-T4 and L-T4 were incubated at a concentration of 1 μM in S1 nuclease (2 U to 20 U), DNase (0.05 U to 1 U), and 90% human serum at 37 °C for various incubation times according to the manufacturers’ protocols or methods that were described in a previous publication [20]. The digestion results were confirmed using 8% denaturing urea (8 M) polyacrylamide gels. All gels were stained in 0.5× TBE buffer with 1× safe pinky (Gendepot, TX, USA) and visualized using an I-MAX gel image analysis system (Core Bio, Seoul, Korea).

### 2.4. Direct Competitive Enzyme-Lined Aptamer Assays (ELAAs) in Which BLI Is Utilized

In a direct competitive assay, ForteBio Octet RED 384^TM^ and streptavidin-coated (SA) sensors (Pall Life Sciences, USA) were used to study interactions between aptamers (D-T4 and L-T4) or negative controls (oligdT and TATA dsDNA) and BPA-HRP conjugate. The buffer recipe, shaking speed during the assay and sensor preparation method were the same as in previous publications [21,22]. Sensors were loaded with biotinylated aptamers/controls (200 nM) and blocked by biocytin (2 µM; to block free streptavidin binding sites). A competition step was performed for incubation with BPA-4-HRP and BPA. In the following step, the signal was amplified by a metal-enhanced DAB substrate. Data were collected and analyzed by Octet analysis software version 8.0. To monitor nonspecific binding to the biosensor, a reference sensor (without loading of biotinylated nucleotides) was included and subjected to the same procedure as the sensors that were loaded with biotinylated nucleic acids. The specificities of the BPA aptamer toward BPA and its analogs were also analyzed. In the BLI assay, 1 ng/mL BPA and its analogs, such as BPB, BP and 6F, in competition with BPA-4-HRP, were used.

### 2.5. Synthesis of AuNPs

AuNPs were synthesized using the citrate reduction method as described previously [23]. Briefly, AuNP synthesis was initiated by the addition of 5 mL of 38.8 mM sodium citrate solution to 50 mL of boiling 1 mM HAuCl_4_ with vigorous stirring. A color change was observed immediately from pale yellow to dark blue and finally to wine red. Boiling was continued for another 10 min, and the reaction flask was incubated at room temperature overnight with stirring. Then, the AuNP solution was filtered through a 0.45 µm syringe filter and stored at 4 °C for further use.

### 2.6. AuNP-Based Colorimetric Detection of BPA

Prior to the assay, the required volumes and concentrations of assay components were optimized under the tested conditions to realize sensitive detection limits. Briefly, 25 µL of 1 µM aptamer in the binding buffer was incubated with 200 µL of AuNPs and 250 µL of triple distilled water for 15 min at room temperature with shaking. Then, various concentrations of 25 µL of BPA were added to the aptamer-coated AuNPs and incubated for another 15 min at room temperature. Next, 15 µL of 0.5 M NaCl was added and incubated for another 5 min to observe the aggregation of AuNPs. Finally, the solution was transferred to a quartz cuvette, and the UV-visible absorbance spectra were measured in the wavelength range between 400–700 nm. The absorbance ratio of A_640_/A_520_ was used for the calibration curve, and the LOD was estimated using 3 × SD of the minimal signal responsive concentration. The assay specificity was also tested with BPA analogs at a fixed concentration of 100 ng/mL.

## 3. Results and Discussion

### 3.1. Design and Characterization of the Spiegelmer

Due to their high specificity and affinity, aptamers that are selected against target molecules have been extensively used as recognition probes in biosensor development. However, as aptamers are D-form biopolymers of natural origin, biopolymers are sensitive to omnipresent degrading enzymes in virtually all biological fluids; thus, stability issues can arise in biological environments. One common strategy for at least partially overcoming this severe limitation is to introduce nonnatural nucleotide analogs (such as 2′-fluoro, 2′-amino, and 2′-O-methyl-pyrimidine RNA) into modified SELEX libraries. Modified nucleotide analogs can increase aptamer ribonuclease resistance, but their use inevitably involves modified enzymes as amplification tools during in vitro selection. Sometimes, the use of modified nucleotide analogs and enzymes is a more laborious process, and the reaction may fail due to the low efficiency of the modified enzymes or harsh working conditions that are difficult to withstand.

**Figure 2:**
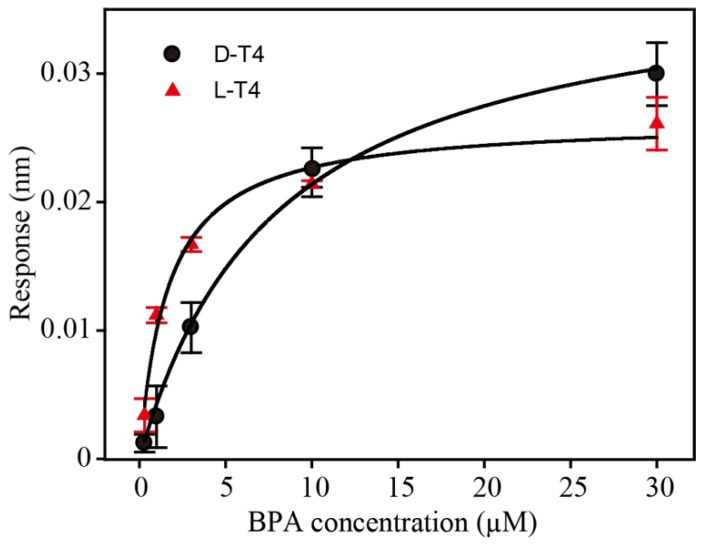
Dissociation constant of the BPA aptamer (D-T4) and its spiegelmer (L-T4). Biolayer interferometry was used to measure the aptamer/spiegelmer binding to BPA (n = 3). The *K_D_* values were estimated as 6.4 and 1.6 µm for D-T4 and L-T4, respectively.

To overcome this challenge, the introduction of stereochemistry and mirror-image oligonucleotides can render aptamers biostable. Generally, if an in vitro selected D-form aptamer binds to its natural target, the enantiomer (L-form aptamer) will bind with the same characteristics to the mirror-image target (unnatural). Due to the homochirality of life and most biochemical compounds, such enantio-ligands would be of limited practical use. If, reciprocally, the SELEX process is carried out against an unnatural target, an aptamer that recognizes the unnatural target will be obtained. Following the principles of stereochemistry, the corresponding mirror-image configuration of this aptamer, namely, the desired L-form aptamer, in turn, recognizes the natural target (see Figure 1). As BPA is a symmetric molecule, the process of synthesizing the mirror-form target can be omitted; hence, the D-form aptamer can be converted directly into an L-form aptamer without the mirror-target synthesis and SELEX process. This provides a very easy and general way to implement a fast selection process for mirror-form aptamers (see Figure 1). To prove our concept, the *K_D_* values of both D and L-form aptamers were tested by biolayer interferometry (Figure 2). The *K_D_* values were estimated as 6.4 and 1.6 µM for D-T4 and L-T4, respectively.

### 3.2. Biostability of the Spiegelmer

**Figure 3:**
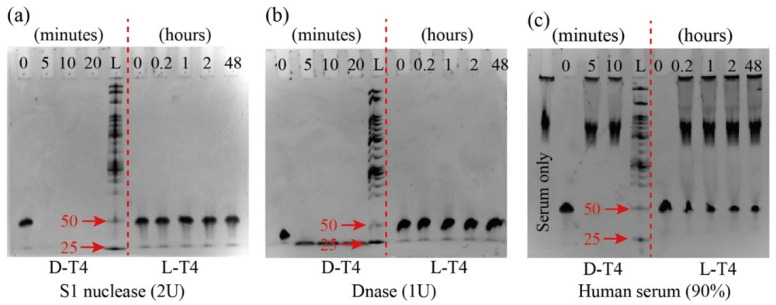
Stability of 48-mer D-T4 (left of the L mark) and 48-mer L-T4 (right of the L mark) under S1 nuclease of 2U (**a**), DNase of 1U (**b**), and human serum of 90% (**c**). Various timelines are also displayed. L denotes the size standard of DNA ladder (25 denotes 25bp and 50 denotes 50bp, respectively).

L-T4 showed exceptional stability against S1 nuclease and DNase in various enzyme units (2U to 20U in S1 nuclease and 0.01U to 1U in S1 DNase). Figure 3 displays the representative feature of its stability when incubated with 2U of S1 nuclease and 1U of DNase for two days (Other concertation of S1 nuclease and S1 DNase were shown in S-2). In comparison, D-T4 degraded in less than five minutes with both enzymes. L-T4 also showed resistance in 90% human serum, whereas D-T4 degraded in less than five minutes (see Figure 3c). The key reason for the nuclease resistance is the configuration of the nucleic acid backbone (the structure is presented in S-1). Naturally occurring nucleases consist of L-amino acids and only accept substrate molecules in the correct chiral configurations (D-form nucleic acids). Consequently, L-form nucleic acids can escape enzymatic recognition and subsequent degradation [12].

### 3.3. Direct Competitive to Detect BPA and Cross-Reactivity

**Figure 4:**
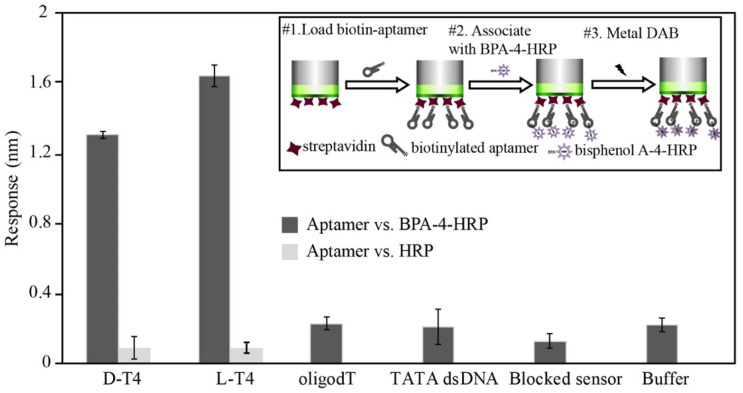
Schematic diagram (upper right-hand inset) and results of the affinity ranking by the SA sensor in the BLI analysis. BPA-HRP conjugate was captured by BPA aptamer on the sensor surface, and subsequent HRP activity was detected by metal DAB.

With BLI, the direct detection of the BPA molecule yields a significantly weaker response due to its low molecular weight, and thus, competition between the BPA-HRP conjugate and BPA was performed in the BLI assay platform (See inset of Figure 4). The target and control sequences that were used in the BLI assay platform are listed in S-1. As shown in Figure 4, the binding response of BPA-HRP was confirmed with both D/L-form aptamers. The HRP background effect was found to be minimal for both aptamers. The specificity of binding was also confirmed by random nucleic acids (oligo dT and TATA dsDNA) and negative sensor or assay buffer.

**Figure 5:**
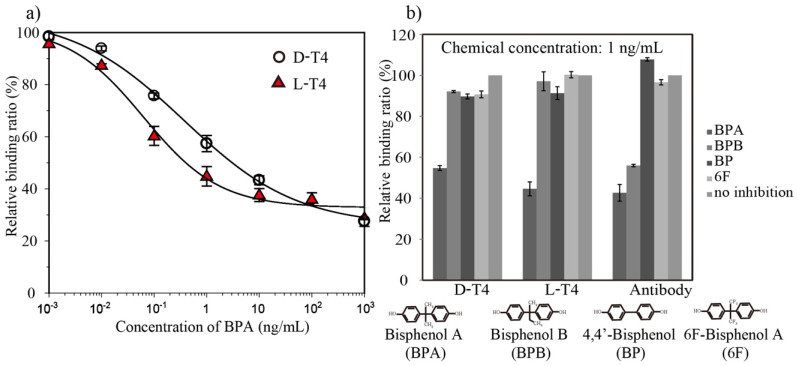
(**a**) A direct competitive ELAA between the BPA-HRP conjugate and free BPA. The BPA aptamers (D-T4 and L-T4) were immobilized on the sensor surface, and subsequent enzyme label detection was performed by BLI. Competition curves were obtained with 200 nM aptamers and a 1/100 dilution of an anti-BPA antibody. The error bars are standard deviations of the mean with n = 3. (**b**) The specificities of the aptamer and antibody for BPA and other analogs. The binding responses for other analogs were measured at the same concentration of 1 ng/mL as BPA.

After confirming the specific binding of BPA-HRP, a direct competitive ELAA was optimized in terms of several factors, such as the aptamer loading concentration and BPA-HRP dilution factor. Later, seven concentrations of BPA (0.001 to 1000 ng/mL) were mixed with BPA-HRP (1/100 dilution) and applied to aptamer-coated biosensor surfaces, and the LODs were calculated to be 0.03 ng/mL and 0.024 ng/mL for D-T4 and L-T4, respectively (Figure 5a). To evaluate the selectivity of the BPA aptamer toward BPA and its analogs, a cross-reactivity analysis was performed at a fixed concentration (1 ng/mL) of BPA and its analogs, which included 6F, BP and BPB, to compete with the BPA-HRP conjugate. These molecules have similar structures to BPA, and their BLI binding response changes are displayed in Figure 5b. In the case of both D-form and L-form aptamers, there was hardly any change in the binding response after the addition of analogs, which suggests that BPA can be easily differentiated when other analogs are present, while the anti-BPA antibody exhibited cross-activity with BPB.

### 3.4. AuNP-Based Colorimetric Detection

**Figure 6:**
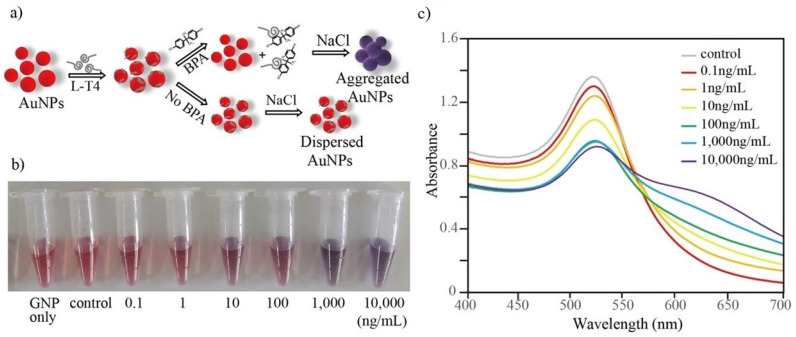
(**a**) A schematic diagram of AuNP-spiegelmer-based BPA detection. (**b**) An image of the color change for the L-T4-AuNP assay. (**c**) UV–vis absorbance spectra of the L-T4-AuNP assay with various concentrations of BPA.

A schematic diagram of the detection method is shown in Figure 6a. Aptamer-mediated salt-induced AuNP aggregation-based colorimetric detection is simple and label-free and has been widely employed for the detection of various small molecules, proteins, and complex cells [24,25,26,27]. Various AuNP-based detection strategies have also been developed against BPA using a D-form aptamer [24,28]. In this study, a similar strategy was applied as a proof of concept for the sensitive detection of BPA using L-T4. L-form DNA/RNA aptamer-based ligands have been successfully used in various bioanalysis methods with several advantages over the D-form [12,29]. Prior to the implementation of the detection method, the volume of AuNPs and the concentration of aptamer that was required to completely coat the surfaces of the AuNPs were optimized. Initially, various volumes (0–50 µL) of 0.5 M NaCl were added to 500 µL of the reaction mixture (which contained 200 µL AuNPs, 250 µL triple distilled water and 50 µL binding buffer) and incubated for 5 min at room temperature to observe the aggregation. Under the tested conditions, 15 µL of 0.5 M NaCl was found to be optimal for aggregating the AuNPs and, thus, was used in further studies (data not shown). Next, the minimum concentration of aptamer that was required to completely capture and to prevent aggregation was analyzed. Among the tested concentrations, 50 µL of 500 nM L-T4 was found to be minimal for preventing the aggregation of AuNPs when tested with 15 µL of NaCl (data not shown).

**Figure 7:**
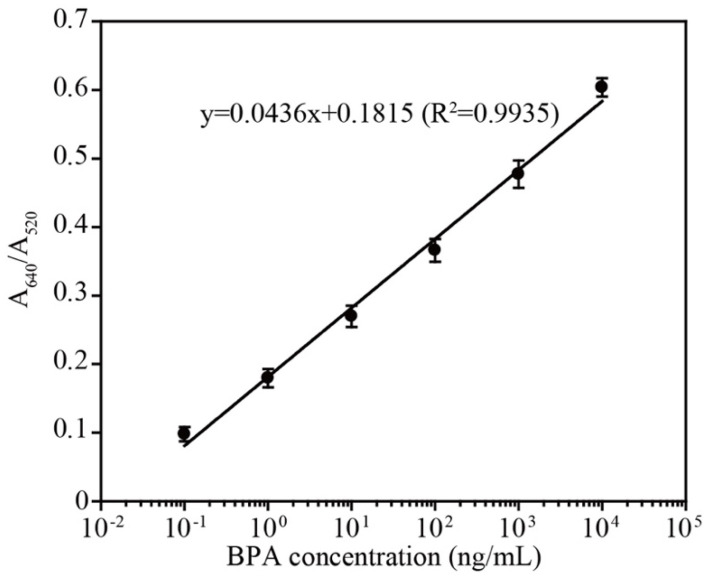
The A_640_/A_520_ ratio versus the BPA concentration. The peak absorbance ratio (A_640_/A_520_) is linear with respect to the logarithm of the BPA concentration (0.1~10,000 ng/mL) with the linear function y = 0.0436x + 0.1815 (R^2^ = 0.9935).

Using the optimized conditions, a colorimetric assay was performed using 25 µL of 1 µM aptamer, 200 µL AuNPs and 250 µL triple distilled water. The detection was initiated by adding 25 µL of various concentrations of BPA in binding buffer at room temperature for 15 min. As shown, dose-dependent aggregation (wine red to purple/blue color) was observed with increased concentrations of BPA (See bottom of Figure 6a). The UV–visible absorbance spectrum also showed a BPA concentration-dependent increase in the absorbance maximum at 640 nm (purple/blue color of aggregated AuNPs), where a decrease in absorbance was observed at 520 nm (wine red color of dispersed AuNPs) (Figure 6b). The calibration curve that was based on the ratio of A_640_/A_520_ showed a linear response from 0.1–10,000 ng/mL BPA as shown in Figure 7. As per the calibration curve, the limit of detection of BPA was estimated to be 0.057 ng/mL. The LOD using L-T4 is correlated with the previously reported LOD values for detection methods that utilized the D-form aptamer [24,28,29]. Furthermore, the specificity of this assay system was also investigated with various BPA analogs. The aptamer showed aggregation only when incubated with BPA, whereas no color change was observed with analog molecules (See Figure S3, Supplementary Materials). The higher stability of the L-form aptamer compared with the D-form may constitute a crucial advantage in the development of various biosensors (see Table 1), especially when tested in complex biological mixtures. Our developed sensor not only showed exceptional stability against digestion enzymes such as S1 nuclease and DNase but also displayed reasonable sensitivity compared with other detection methods (as in the Table 1), which explored rapid but biostable environmental BPA biosensors.

## 4. Conclusions

The incorporation of aptamers into aptasensor development for detection in a harsh environment is severely limited by their low biostability. We combined the principles of chirality with the powerful SELEX process, which has led to the development of highly biostable and extremely specific agents, namely, spiegelmers, and demonstrated the ability of our aptasensor platform to act as a precise detection system for bisphenol A (BPA). In this study, we firstly showed the long-term biostability of the spiegelmer aptamer of BSA against the enzymes which degrades the nucleic acid. When mixed with S1 nuclease, DNase, or human serum, the spiegelmer aptamer shows excellent stability which is not degraded compared with the D-form aptamer. Such an excellent biostability makes it possible to use this type of aptamer as a probe of biosensors for monitoring environments such as water recourse. Secondly, we set up sensing formats of ELAA and an AuNP-based colorimetric as a proof of principle using the spiegelmer aptamer of BSA.

The aptasensor provided an excellent sensitivity to BPAs with LODs of 0.024 ng/mL and linearity from 100 pg/mL to 10 mg/mL with 0.99 of R square. In addition, our simplified Au-colorimetric assay showed feasible sensitivity and selectivity providing possible application of our system to in situ field tests. Further investigation into the potential of spiegelmers is in progress, and mirror-image oligonucleotides are expected to have applications in on-site environmental monitoring.

## Figures and Tables

**Table 1:** Various BPA biosensors with aptamer as probe.

Categories	Detection Methods	LOD	Dynamic Range	Ref.
Carbon nanotube transistor sensor	SwCNT-FET capacitance based sensor	1 pM	-	[30]
Capacitance sensor	AAO-based capacitance sensor	1 nM	1 nm~100 nm	[31]
Colorimetric sensor	GNA and aptamer	0.1 ng/mL	0.1~100 ng/mL	[24]
Colorimetric sensor	GNA and aptamer	1 pg/mL	1 pg/mL~1 mg/mL	[32]
Fluorescent sensor	Small GNP’s with functionalized aptamer	0.1 ng/mL	1~10,000 ng/mL	[33]
Electrochemical sensors	Competitive cDNA aptasensors	0.284 pg/mL	0.284~1000 pg/mL	[34]
Electrochemical sensor	DPV using AuNP/MWCNT/GCE	4 nM	0.01~0.7 µM	[35]
Electrochemical sensor	DPV using AuNP–rGO–MWCNTs/GCE	1 nM	5 nM~20 µM	[36]
This method	GNP with L-form DNA aptamer	0.1 ng/mL	0.1 ng/mL~1 mg/mL	

## Data Availability

Not applicable.

## Supplementary Materials

The following supporting information can be downloaded at: www.mpdi.com/xxx/s1, Figure S1: An aptamer against BPA (D-T4) and its sequence were designed by our previous paper [18]. L-form aptamer (L-T4) was synthesized based on D-T4 aptamer. Negative controlled nucleic acid such as oligo dT and TATA dsDNA) were synthesized and their sequence were also printed. The secondary structures of the aptamer and spiegelmer were predicted by *Mfold*. The nucleotides are numbered every 10 bases, and the 5’ and 3’ termini are labeled. The aptamer and spiegelmer share the same nucleotide sequence (left) but differ in terms of sugar backbone structure (right), Figure S2: (a) the stability test of L-4 aptamer against S-1 nuclease units at 2U, 4U, 10U, and 20U, respectively. (b) the stability test of L-4 aptamer against TURBO^TM^ DNase units at 0.05U, 0.25U, 0.5U, and 1U, respectively, Figure S3: The specificity of L-T4 with BPA analogs (BPB, BP and 6F) at 100 ng/ml for a AuNP-based assay. An image of the color change for the L-T4-AuNP specificity assays were presented on the right side of the figure.
