# Supplementary material for: Nonbiodegradable Spiegelmer-Driven Colorimetric Biosensor for Bisphenol A Detection
Source: Biosensors (Basel). 2022 Oct 12;12(10):864. doi: 10.3390/bios12100864 (PMC9599196; doi:10.3390/bios12100864)
Supplement: Supplementary file 1 [file biosensors-12-00864-s001.zip › biosensors-1935155-supplementary.pdf]

Supporting Information

Nonbiodegradable Spiegelmer-Driven Colorimetric Biosensor for Bisphenol A Detection

Shuo Ren [1], Samuel Cho [1], Ruixan Lin [2], Vinayakumar Gedi [3], Sunyoung Park [4], Chul Woo Ahn [5,6], Dong-Ki Lee [3], Min-Ho Lee [2] SangWook Lee [1,7,*] and Soyoun Kim [1,8,*]

[1] PCL Inc., Seoul 05854, Korea
[2] School of Integrative Engineering, Chung-Ang University, Seoul 06974, Korea
[3] Global Research Laboratory (GRL) for RNAi Medicine, Department of Chemistry, Sungkyunkwan, University, Suwon 16419, Korea
[4] Gangnam Biomedical Research Center, Yonsei University College of Medicine, Seoul 06273, Korea
[5] Division of Endocrinology Department of Internal Medicine, Yonsei College of Medicine, Seoul 03722, Korea
[6] Severance Institute for Vascular and Metabolic Research, Yonsei University College of Medicine, Seoul, Korea
[7] Research Center for Advanced Science and Technology (RCAST), The University of Tokyo, Tokyo 153-8904, Japan
[8] Convergence Research Institute, Korea University, Seoul 02841, Korea
* Correspondence: swlee@pclchip.com (S.L.); soyouny9@korea.ac.kr (S.K.)

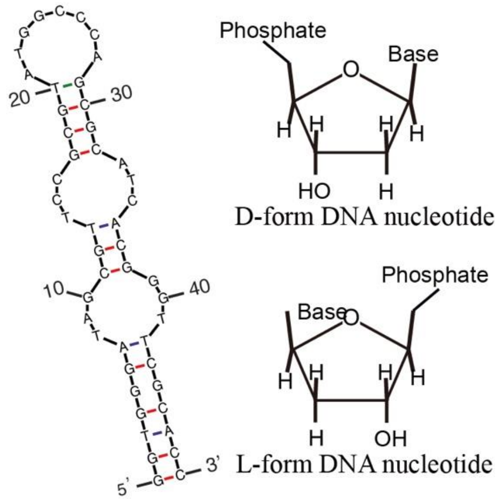

aptamer	sequence
D-T4	5′-GGTGGGATAGCGTTCCGCGTATGGCCCAGCGCATCACGGGTTCGCACC-3'
L-T4	5′-*GGTGGGATAGCGTTCCGCGTATGGCCCAGCGCATCACGGGTTCGCACC*-3'
oligo dT	5'-TTTTTTTTTTTTTTT-3'
TATA dsDNA	5'-GGGAATTCGGGCTATAAAAGGGGGATCCGG-3′<br>3'-CCCTTAAGCCCGATATTTTCCCCCTAGGCC-5'

Figure S-1. An aptamer against BPA (D-T4) and its sequence were designed by our previous paper [18]. L-form aptamer (L-T4) was synthesized based on D-T4 aptamer. Negative controlled nucleic acid such as oligo dT and TATA dsDNA) were synthesized and their sequence were also printed. The secondary structures of the aptamer and spiegelmer were predicted by *Mfold.* The nucleotides are numbered every 10 bases, and the 5' and 3' termini are labeled. The aptamer and spiegelmer share the same nucleotide sequence (left) but differ in terms of sugar backbone structure (right).

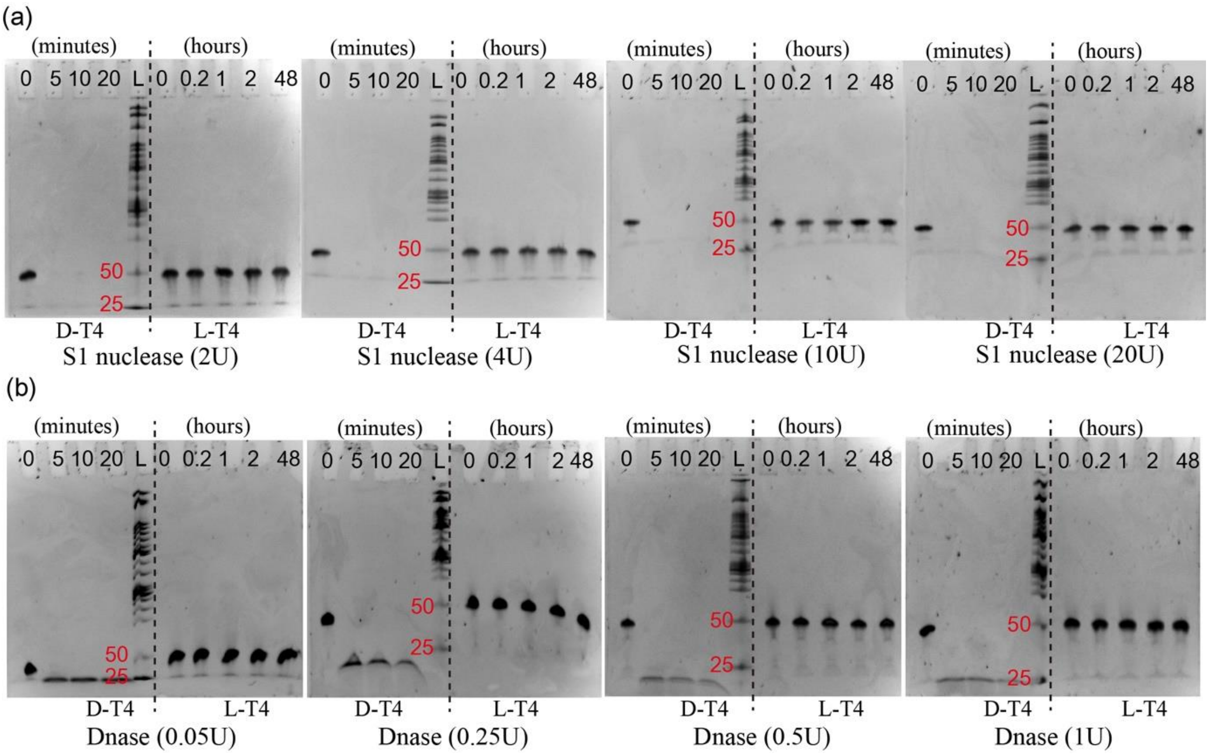

Figure S-2 a) The stability test of L-4 aptamer against S-1 nuclease units at 2U, 4U, 10U, and 20U, respectively. b) The stability test of L-4 aptamer against TURBO^{TM} DNase units at 0.05U, 0.25U, 0.5U, and 1U, respectively.

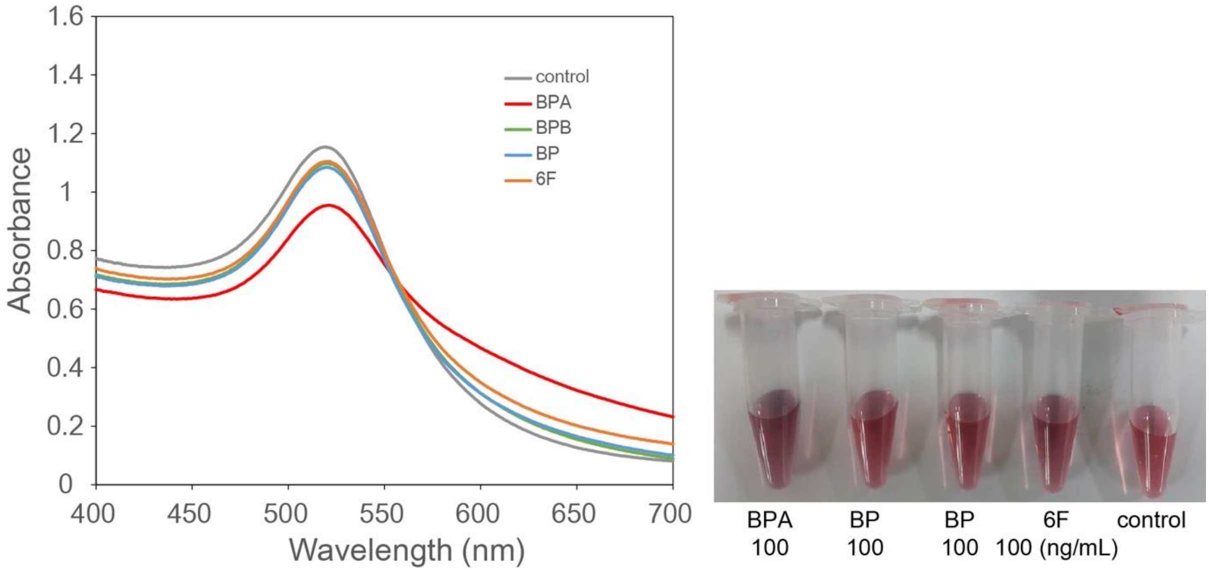

Figure S-3. The specificity of L-T4 with BPA analogs (BPB, BP and 6F) at 100 ng/ml for a AuNP-based assay. An image of the color change for the L-T4-AuNP specificity assays were presented on the right side of the figure.
